# Microbial Transglutaminase Increases Uptake and Translocation of Gliadin Peptides in the Human Intestinal Epithelium

**DOI:** 10.1002/mnfr.70197

**Published:** 2025-08-11

**Authors:** Sebastian Stricker, Jan De Laffolie, Klaus‐Peter Zimmer, Silvia Rudloff

**Affiliations:** ^1^ Department of General Pediatrics and Neonatology Justus‐Liebig‐University Giessen Giessen Germany; ^2^ Department of Nutritional Science Justus‐Liebig‐University Giessen Giessen Germany

**Keywords:** celiac disease, gliadin peptide, microbial transglutaminase, peptide transport, transglutaminase 2

## Abstract

Transglutaminase 2 (TG2) plays an essential role in the pathogenesis of celiac disease (CD) by modifying gliadin peptides. Additionally, microbial transglutaminase (mTG) was implicated in CD pathogenesis since it modifies gliadin peptides in the same way. mTG is used as a technical aid in the production of food items and might also be released by the intestinal microbiota. We aimed to characterize the interaction of mTG with different gliadin peptides and its effect on the epithelial and mucosal transport.We compared mTG‐ and TG2‐mediated crosslinking of gliadin peptides using indirect enzyme‐linked immunosorbent assay (ELISA) and immunoblot. Epithelial uptake of gliadin peptides P56‐88 and P31‐49 by Caco‐2 cells and human duodenal biopsies was quantified by fluorometry and immunofluorescence microscopy. mTG had a much higher affinity towards different gliadin peptides than TG2. mTG significantly increased the uptake of gliadin peptide P56‐88 and P31‐49 by Caco‐2 cells by nearly 10 times. Finally, mTG increased the uptake and colocalized with gliadin peptide P56‐88 in the human duodenal mucosa. DmTG‐mediated enzymatic modification of gliadin peptides is evident and influences the epithelial uptake of potentially detrimental gliadin peptides. The uncritical use of mTG in food production might therefore pose a potential threat for consumers.

## Graphical abstract

The bacterial enzyme microbial transglutaminase (mTG) is widely used in the food industry as a technological aid to improve textural properties of a multitude of food products. mTG mimics the enzymatic function of human transglutaminase 2 (TG2) and may play a role in the pathogenesis of celiac disease. Here, we demonstrate that mTG has a high affinity to different gliadin peptides and significantly increases their uptake by intestinal epithelial cells and the human duodenal mucosa.

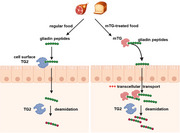

**Abbreviations**
5BP: 5‐biotinamidopentylamine
5‐FAM: 5‐carboxyfluorescein
CD: celiac disease
DTT: dithiotreitol
EDTA: ethylenediamine tetraacetic acid
ELISA: enzyme‐linked immunosorbent assay
ESPGHAN: European Society for Paediatric Gastroenterology, Hepatology and Nutrition
GFD: gluten‐free diet
HLA: human leukocyte antigen
HRP: horseradish peroxidase
mTG: microbial transglutaminase
PBS: phosphate buffered saline
PTG: pepsin/trypsin digested gliadin
TEER: transepithelial electrical resistance
TG2: transglutaminase 2
TMB: tetramethylbenzidine
WGA: wheat germ agglutinin

## 1 Introduction

Celiac disease (CD) represents a chronic inflammatory disease with increasing incidence in the Western world [1]. It is triggered by the ingestion of specific prolamines (gliadin, hordein, and secalin) in individuals carrying the genetic predisposition (human leukocyte antigen [HLA]‐DQ2/8). Transglutaminase 2 (TG2) is the recognized autoantigen of the disease and plays a key role in the pathogenesis [2]. TG2 deamidates specific glutamine residues within the 33‐mer α‐gliadin peptide (P56‐88; PQLP → PELP), which markedly increases its affinity to the HLA‐DQ2 receptor of antigen‐presenting cells [3]. This enzymatic modification is a precondition for the subsequent HLA‐II‐mediated antigen presentation, which finally results in the Th1‐mediated immune response and damage of the intestinal mucosa [4, 5, 6]. Recent evidence also indicated that cellular TG2 contributes to the immunopathogenesis of CD by promoting endocytosis of potentially harmful gliadin peptides by the gastrointestinal epithelium [7, 8, 9, 10]. In addition to P56‐88, the gliadin peptides P31‐43 and P31‐49 were suggested to have direct “toxic” effects on intestinal epithelial cells, duodenal biopsies, and cultivated organoids [11, 12, 13, 14, 15, 16].

The increasing incidence of CD and the fact that only a small proportion of genetically predisposed individuals actually develop the disease suggest that other exogenous factors contribute. In this context, microbial transglutaminase (mTG) was implicated to play a role in CD [17, 18, 19, 20]. This bacterial enzyme mimics the enzymatic function of TG2 although it has no sequence homology [21, 22, 23, 24]. It works calcium‐independent and has a much broader substrate specificity. In contrast to other transglutaminases, mTG also mediates the deamidation of the 33‐mer α‐gliadin peptide similar to TG2 [17, 25]. mTG is widely used in the food industry as a technological aid to improve textural properties of a multitude of food products [26, 27, 28]. Even though the usage of mTG has raised some safety concerns, especially for people carrying the genetic predisposition for CD, there is currently no regulation or declaration of mTG‐treated foods in the European Union [29, 30]. In addition to food products, the intestinal microbiota might also represent a source of active mTG in the gut lumen [24]. The interaction of mTG with different gliadin peptides and the impact of mTG on gliadin peptide uptake have been hardly addressed in the past. Therefore, we aimed to further characterize the interaction between mTG and different gliadin peptides and its impact on the epithelial and mucosal transport of immunogenic and toxic gliadin peptides.

## 2 Materials and Methods

### 2.1 Patients’ Characteristics

The study was approved by the local ethics committee (reference number 146/16), and informed consent was obtained for every patient. Duodenal biopsies were obtained from five CD patients and five control patients with gastritis and esophagitis during clinically indicated upper gastrointestinal endoscopy. CD was diagnosed according to current guidelines of the European Society for Paediatric Gastroenterology, Hepatology and Nutrition (ESPGHAN) [31] (Table 1).

**TABLE 1:** Patients’ characteristics.

Patient	Group	Sex	Age	Anti‐TG2‐IgA (IU/mL)	Marsh‐grade	Remarks
1	Control	F	14	9.6	0	Gastritis Type C
2	Control	M	17	<2	0	Reflux esophagitis
3	Control	M	17	<2	0	Gastritis Type C, Crohn's Disease
4	Control	M	15	<2	0	Eosinophilic esophagitis
5	Control	F	16	<2	0	Gastritis Type C
6	CD	F	10	>200	3a	—
7	CD	F	14	>200	3b	—
8	CD	F	10	>200	3c	—
9	CD	M	14	111	3a	GFD
10	CD	F	13	2.9	0	GFD

*Note*: Cutoff anti‐TG2‐IgA > 20 IU/mL.

Abbreviations: F, female; GFD, gluten‐free diet; M, male.

### 2.2 In Vitro Transamidation of Gliadin Peptides by mTG and TG2

TG2 (10 nM, T022, Zedira, Darmstadt, Germany) or mTG (10 nM, T001, Zedira) were immobilized on a 96‐well plate (Sarstedt, Nümbrecht, Germany) in 50 mM Tris, 1 mM ethylenediamine tetraacetic acid (EDTA), 5 mM calcium chloride (pH 7.5) buffer containing 10 mM dithiotreitol (DTT, Merck KGaA, Darmstadt, Germany). After blocking with 2% bovine serum albumin (BSA, Carl Roth GmbH, Karlsruhe, Germany), the transglutaminase inhibitors ERW1041 (5095220001, Sigma–Aldrich, Darmstadt, Germany) and mTG‐blocker (C102, Zedira, each 100 µM) were applied according to protocol. Immobilized transglutaminases were incubated with biotin‐conjugated gliadin peptides or pepsin/trypsin digested gliadin (PTG, Table 2). Detection of crosslinked biotinylated gliadin peptides and PTG was done using horseradish peroxidase (HRP)‐conjugated streptavidin (1:2500, 405210, Biolegend, San Diego, CA, USA) or a specific gliadin antibody (1:1000, clone XGY4, Zedira) and the corresponding secondary HRP‐conjugated antibody (1:1000; sc‐516102, Santa Cruz Biotechnology, Dallas, TX, USA). The HRP‐substrate tetramethylbenzidine (TMB, T0440, Sigma–Aldrich, Darmstadt, Germany) was added, and photometric quantitation at 655 nm was performed after 15 min using a Clariostar Plus microplate reader (BMG Labtech, Ortenberg, Germany).

**TABLE 2:** Gliadin peptides used for ELISA and immunoblot.

Substance	Sequence	ELISA (µg/mL)	Immunoblot (µg)	Company
P31‐49‐biotin	Biotin‐LGQQQPFPPQQPYPQPQPF	10	2	Biosynth, Lelystad, Netherlands
P56‐68‐biotin	Biotin‐LQLQPFPQPQLPY	10	1	Biosynth, Lelystad, Netherlands
P56‐88‐biotin	Biotin‐LQLQPFPQPQLPYPQPQLPYPQPQLPYPQPQPF	10	1	Biosynth, Lelystad, Netherlands
P57‐73E	QLQPFPQPELPYPQPQS	10	10	Lifetein, Somerset, NJ, USA
P229‐246‐biotin	Biotin‐LPQFEEIRNLALQTLPAM	10	2	Biosynth, Lelystad, Netherlands
PTG (P004)	—	10	0.5	Zedira, Darmstadt, Germany

### 2.3 Immunoblot

TG2 or mTG (1 µM) was incubated with gliadin peptides (Table 2) with or without ERW1041 as TG2 blocker or the mTG‐blocker (100 µM) in Tris buffer. Biotinylated gliadin peptides or PTG was added to the solution at the indicated concentrations, and incubation was performed for 30 min at 37°C. The reaction was stopped by placing the samples on ice. Detailed description of the conditions for electrophoresis and blotting have been published elsewhere [7]. After blocking the membrane for 1 h with 5% nonfat dry milk in phosphate buffered saline (PBS) + 2.5% tween, incubation with the primary antibody was performed at 4°C overnight (Table 3). The next day, incubation with the corresponding HRP‐conjugated secondary antibody was done. The crosslinking of biotinylated gliadin peptides was detected by streptavidin‐HRP alone. Visualization was done with the Super‐Signal West Femto kit (34096, Thermo Fisher Scientific, Langenselbold, Germany) for 5 min. Image processing and quantitation were performed using a ChemiDoc XRS+ imager and Image Lab software (BioRad Laboratories, Feldkirchen, Germany).

**TABLE 3:** Primary antibodies used in this study for immunoblot experiments.

Target	Antibody	Host/clonality	Dilution	Company
Gliadin	XGY4	Mouse/monoclonal	1–2500 PBST with 5% nonfat dry milk	Zedira, Darmstadt, Germany
mTG	A145	Rabbit/polyclonal	1–10 000 in PBST with 5% nonfat dry milk	Zedira, Darmstadt, Germany
Biotin	—	Streptavidin‐HRP	1–2500 in PBST with 5% nonfat dry milk	Biolegend, San Diego, CA, USA
TG2	D11A6	Rabbit/monoclonal	1–2500 in TBST with 5% BSA	Cell Signaling Technology, Leiden, The Netherlands

### 2.4 Cell Culture

All cell culture experiments were performed with the human intestinal epithelial cell line Caco‐2 (ATCC HTB‐37). The cells were cultured at 37°C with 5% CO_2_ and 95% humidity in DMEM (41965‐039, Thermo Fisher Scientific) supplemented with 1% penicillin–streptomycin (15140‐122, Thermo Fisher Scientific), 1% nonessential amino acids (11140‐050, Thermo Fisher Scientific), 1% sodium pyruvate (11360‐039, Thermo Fisher Scientific), and 10% heat‐inactivated fetal bovine serum (A5256701, Thermo Fisher Scientific). The culture medium was changed every two to three days and cells were passaged at 80 % confluence using TrypLE express (12605010, Thermo Fisher Scientific). Cells were seeded at a density of 4 × 10^4^ per cm^2^ onto tissue culture‐treated plates.

### 2.5 Cell Viability of Caco‐2 Cells

The effect of transglutaminases on the viability of Caco‐2 cells was investigated using the resazurin‐based PrestoBlue HS assay (P50200, Thermo Fisher Scientific) as described elsewhere [7]. Details are provided in the Supporting Information.

### 2.6 Extracellular Crosslinking of 5BP by Transglutaminases

Confluent Caco‐2 cell monolayers were incubated with the transglutaminase substrate EZ‐link pentylamine‐biotin (5‐biotinamidopentylamine [5BP], 500 µM, 21345, Thermo Fisher Scientific) with or without mTG or TG2 (100 nM) in Tris buffer for 1 h. Corresponding transglutaminase inhibitors ERW1041 and mTG‐blocker were added to the solution at the indicated concentration. After fixation with 4% paraformaldehyde and blocking with 5% BSA, cells were incubated with streptavidin‐Alexa Fluor488 (S11223, 4 µg/mL, Thermo Fisher Scientific). Fluorometric quantitation was conducted using a FLUOstar Optima microplate reader (BMG Labtech, Ortenberg, Germany). For immunofluorescence microscopy, nuclei were stained with Hoechst dye (1:1000 in PBS, 44442, Thermo Fisher Scientific) for 10 min. Microscopy was done using a confocal laser scanning microscope (TE2000‐E, Nikon) and 20x Plan Apo (NA 1.41) objective. Image processing and quantitation were performed using ImageJ [32].

### 2.7 Epithelial Transport of Gliadin Peptides in the Presence of mTG

Caco‐2 cell monolayers were incubated with either 5‐carboxyfluorescein‐ (5‐FAM, BioCat GmbH, Heidelberg, Germany) or biotin‐conjugated (Biosynth, Lelystad, Netherlands) gliadin peptides P56‐88 and P31‐49 at concentrations of 40 and 25 µg/mL corresponding to a dose of 10 µM. The effect of mTG or TG2 (100 nM) on epithelial peptide uptake was investigated by simultaneous incubation with gliadin peptides in Tris buffer for 1 h at 37°C. The administered dosages for mTG and gliadin peptides in cell culture and ex‐vivo experiments were based on the current literature [12, 14, 26, 27, 33]. Where indicated, the specific inhibitors ERW1041 and mTG blocker (each 100 µM) were added to the incubation solution. For the detailed description see the Supporting Information.

### 2.8 Transepithelial Electric Resistance

Cultivation of Caco‐2 cells on semi‐permeable transwell supports (662640, ThinCert, culture surface: 33.6 mm^2^, pore size: 0.4 µm, Greiner Bio One, Pleidelsheim, Germany) has been described in detail elsewhere [7]. Complete medium exchange was done the day before the experiment (apical 180 µL, basal 500 µL). mTG was diluted in 20 µL medium at the indicated concentrations and added to the apical compartment. Transepithelial electrical resistance (TEER) was measured with a volt‐ohm meter (Millipore, Schwalbach, Germany). Monolayers with an insufficient TEER (<450 Ω/cm^2^) were omitted. Incubation was performed and TEER was measured at the timepoints 0, 1, 2, 3, 6, and 24 h. TEER values of mTG‐treated Caco‐2 cells were normalized against cells only incubated with medium.

### 2.9 Mucosal Uptake of P56‐88 in the Presence of mTG

Duodenal biopsies were obtained during clinically indicated esophagogastroduodenoscopy. Tissue samples were placed in one layer between 1.5 mm circular aperture sliders (P2307C, Physiologic Instruments, Rena, NV, USA). Incubation was performed for 30 min at 37°C with DMEM/F12 (Thermo Fisher Scientific) medium supplemented with biotinylated P56‐88 (20 µM) with or without mTG (1 µM). Detection of mTG in tissue sections was done using a specific primary antibody (A145, 1:200, Zedira) and the corresponding Alexa Fluor555‐conjugated secondary antibody (goat‐anti‐rabbit, 1:200, Thermo Fisher Scientific). Biotinylated P56‐88 was detected by Alexa Fluor488‐conjugated streptavidin (4 µg/mL, Thermo Fisher Scientific). Microscopic analysis was done using a Leica DMi8 fluorescence microscope (Leica, Wetzlar, Germany) and a 63x (NA 1.3) oil objective. Three images per condition were analyzed using ImageJ [32].

### 2.10 Statistics

Statistical analysis was performed using GraphPad Prism 9 (GraphPad Prism Software Inc., San Diego, CA, USA). Student's unpaired two‐tailed *t* test (with Welch correction where appropriate) or Mann–Whitney *U* test was used where appropriate. *p* values less than 0.05 were considered significant.

## 3 Results

### 3.1 mTG Has a Higher Crosslinking Activity on Different Gliadin Peptides Compared to TG2

**FIGURE 1:**
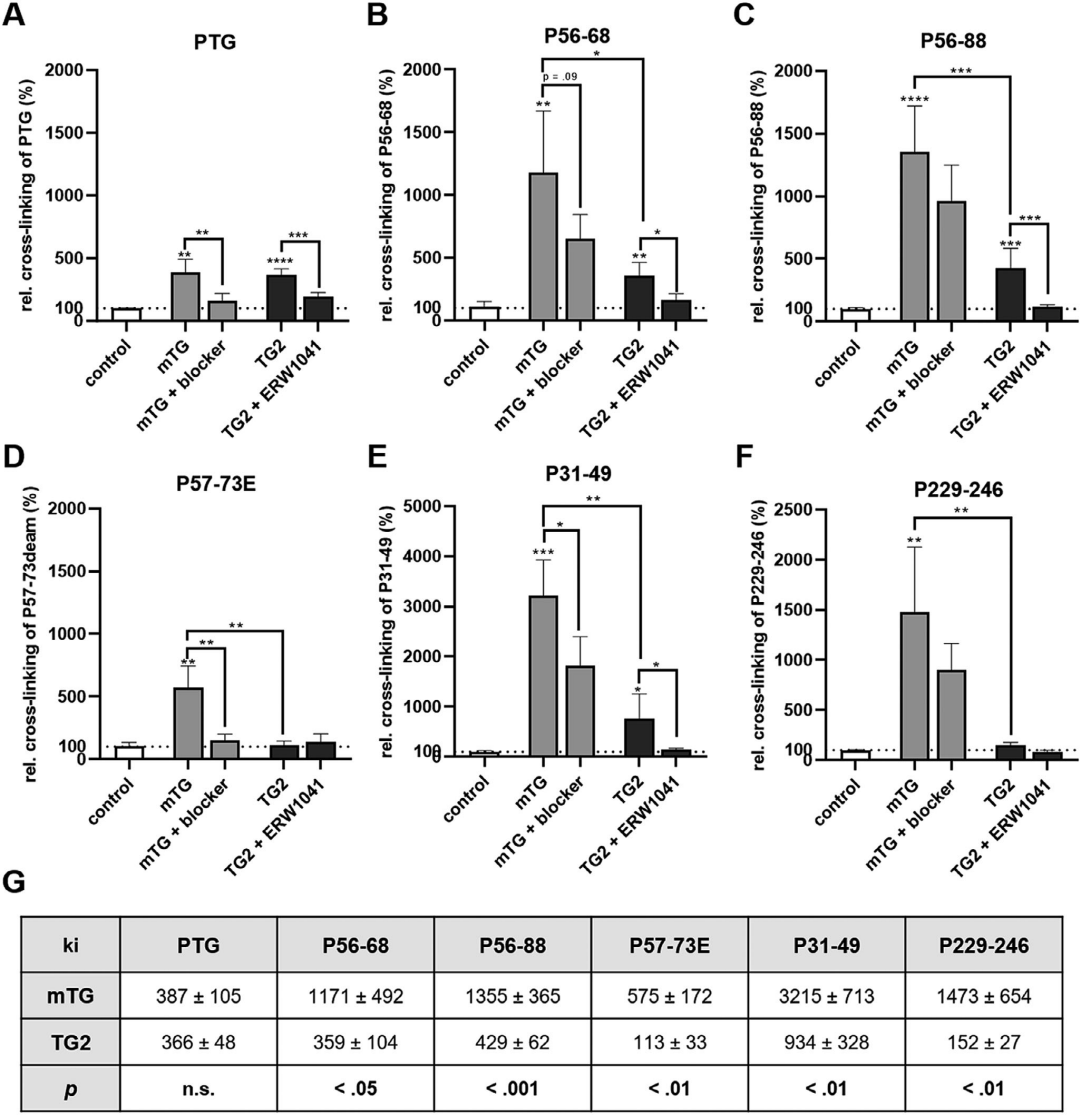
In‐vitro crosslinking of gliadin peptides by mTG and TG2. Transglutaminase‐mediated crosslinking of pepsin/trypsin‐digested gliadin (PTG) (A) and different gliadin peptides (B–F) was investigated by photometry. (G) Crosslinking affinity of mTG and TG2 towards different gliadin peptides. Normalization was performed against a condition, where no transglutaminases were applied (*n* = 4 with two technical replicates per experiment). All data are shown as mean ± SD. Statistical significance was tested with the Student's *t* test. * *p* < 0.05, ** *p* < 0.01, *** *p* < 0.001, **** *p* < 0.0001. mTG, microbial transglutaminase; SD, standard deviation; TG2, transglutaminase 2.

We used an indirect enzyme‐linked immunosorbent assay (ELISA) to investigate the crosslinking of different gliadin peptides by mTG and TG2. First, we observed a similar crosslinking of PTG by both transglutaminases (Figure 1A). Addition of the specific inhibitors mTG‐blocker and ERW1041 significantly reduced crosslinking of PTG proving that the enzymatic function of both transglutaminases mediates this process (Figure 1A). Next, we investigated the crosslinking of the potentially immunogenic gliadin peptides P56‐68 and P56‐88, which contain the specific target sequence (PQLP) for TG2. Both transglutaminases transamidated P56‐68 and P56‐88, but mTG was significantly more efficient than TG2 (Figure 1B, C, G). To address the question, whether deamidation of the glutamine‐containing sequence (PQLP → PELP) affects crosslinking of the corresponding peptides, we used a deamidated gliadin peptide (P57‐73E). In contrast to the nondeamidated peptides P56‐68 and P56‐88, TG2 did not crosslink this modified gliadin peptide (Figure 1D). mTG transamidated P57‐73E, but the effect was much less pronounced compared to the peptides P56‐68 and P56‐88 (Figure 1D). We then investigated the crosslinking of gliadin peptides lacking the target sequence PQLP. Both transglutaminases crosslinked the “toxic” gliadin peptide P31‐49, but again, mTG had a significantly higher affinity than TG2 (Figures 1E, G). Finally, we analyzed crosslinking of the control gliadin peptide P229‐246 with no known immunogenic or “toxic” effects related to CD. TG2 crosslinked the control peptide, but to a much lesser extent compared to CD‐related gliadin peptides (Figures 1F, G). mTG‐mediated crosslinking was significantly stronger compared to TG2 (Figures 1F, G). In total, mTG crosslinking activity was significantly higher and exceeded TG2‐mediated crosslinking by 3–10 times (Figure 1G).

### 3.2 Gliadin Peptides Are Crosslinked to TG2 to a Higher Extent than to mTG

**FIGURE 2:**
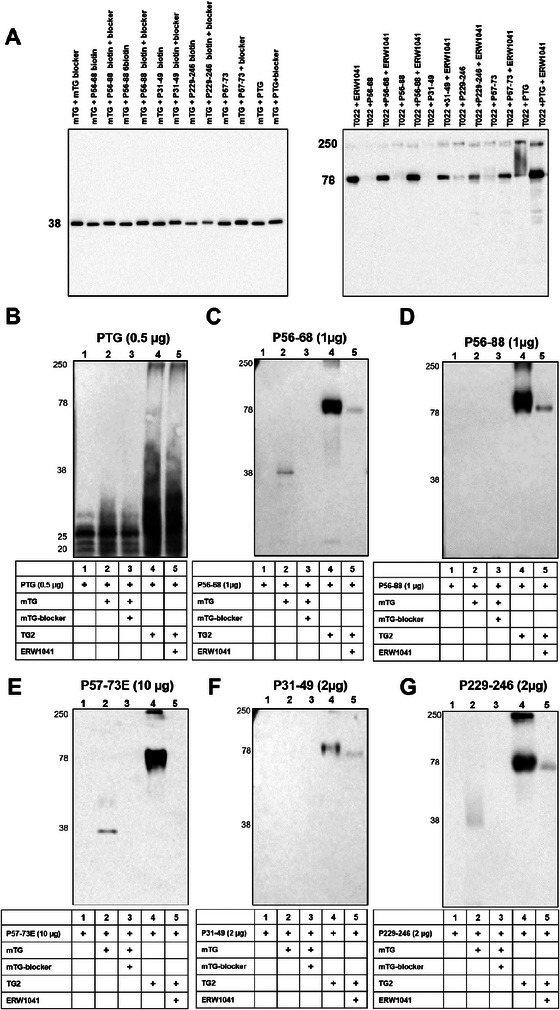
Immunoblotting of gliadin‐transglutaminase complexes. (A) mTG and TG2 were detected by specific antibodies after incubation with gliadin peptides with or without mTG blocker or ERW1041. (B–G) Crosslinking of gliadin peptides was visualized by specific antigliadin‐antibody (PTG, P57‐73E) and the corresponding HRP‐conjugated secondary antibody or HRP‐conjugated streptavidin (biotinylated peptides, P56‐68, P56‐88, P31‐49, P229‐246). *n* = 3, representative immunoblots are shown. HRP, horseradish peroxidase; mTG, microbial transglutaminase; PTG, pepsin/trypsin digested gliadin;TG2, transglutaminase 2.

To further investigate whether both transglutaminases tend to crosslink gliadin peptides with each other, we performed immunoblotting experiments. Gliadin peptides were incubated in the presence of mTG or TG2 with or without their specific inhibitors. Incubation of mTG with different gliadin peptides did not alter the molecular weight of the mTG‐positive band or the affinity of the antibody directed against mTG (Figure 2A). This observation suggested that mTG does not crosslink gliadin peptides with each other. Incubation with the mTG‐blocker, however, resulted in a slightly increased molecular weight indicating a covalent binding of the inhibitor to mTG (Figure 2A). In contrast, TG2 crosslinked the different gliadin peptides with themselves, which was indicated by the presence of high molecular weight bands. This effect was prevented by the addition of ERW1041 suggesting the enzymatically mediated formation of TG2‐gliadin complexes (Figure 2A). To characterize the formation of transglutaminase‐gliadin complex in more detail, we detected the different gliadin peptides with a specific monoclonal antibody (non‐biotinylated peptides) or streptavidin‐HRP (biotinylated peptides). When PTG was treated with both transglutaminases, we observed multiple higher molecular weight bands, indicating the formation of complexes (Figure 2B). This observation was much more pronounced in samples treated with TG2. Coincubation with transglutaminase inhibitors partially reduced this effect (Figure 2B). Using streptavidin‐HRP or a specific antigliadin‐antibody, we detected crosslinking of all peptides to TG2 (78 kDa band) and the presence of high molecular weight complexes (250 kDa band, Figure 2C–G). The addition of the inhibitor ERW1041 significantly reduced transamidation of all gliadin peptides to TG2. Treatment with mTG, however, only revealed a weak band at 38 kDa, corresponding to a low grade of crosslinked peptides (Figures 2C–G). Altogether, TG2 crosslinked gliadin peptides and produced high molecular weight complexes, whereas mTG had a much lower effect.

### 3.3 mTG and TG2 Mediate Crosslinking of Transglutaminase Substrates to the Cell Surface

**FIGURE 3:**
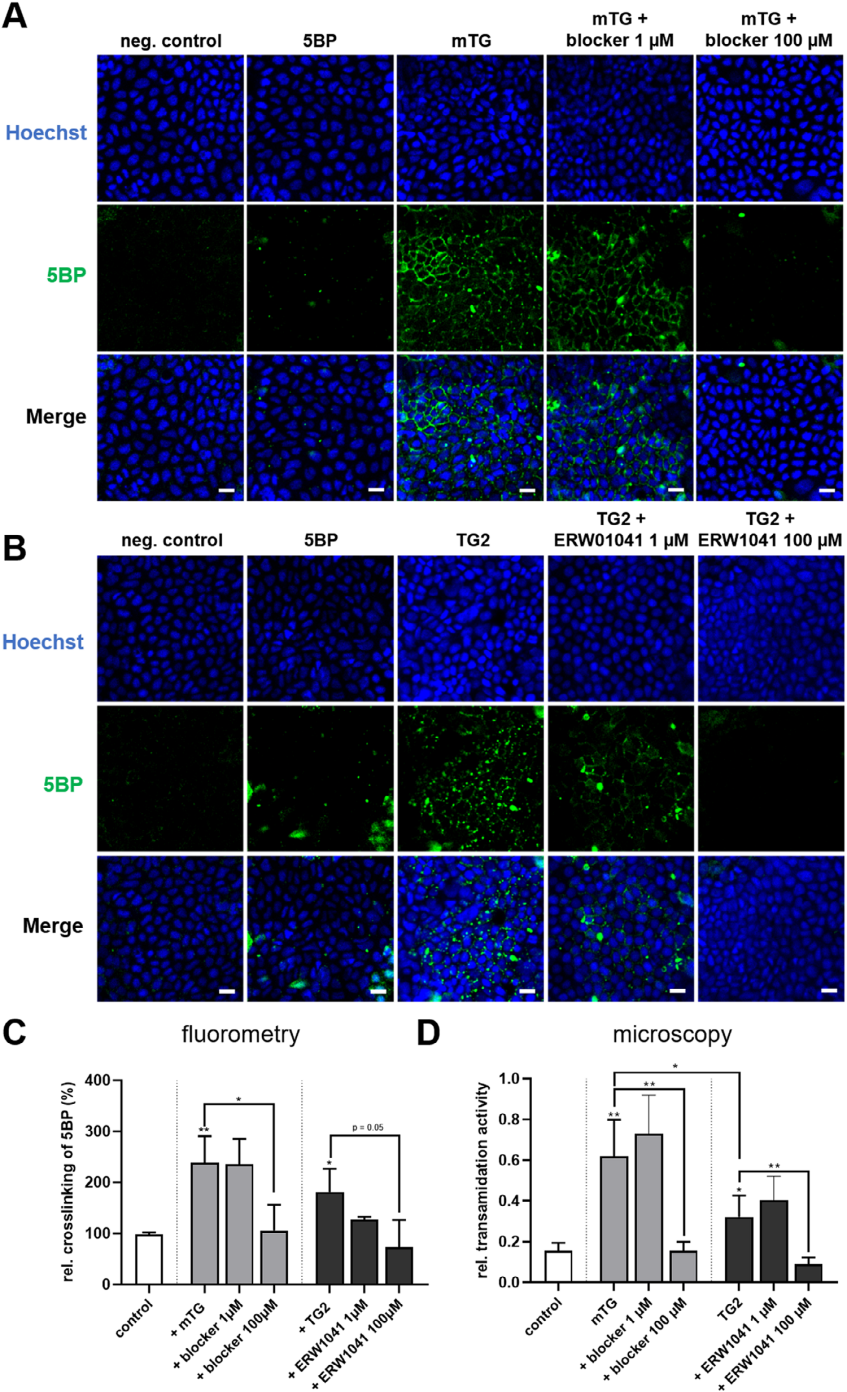
Crosslinking of 5BP to the cell surface of Caco‐2 cells by mTG and TG2. (A, B) Representative confocal microscopic images of Caco‐2 cells incubated with 5BP in the presence of mTG and TG2. (C) Fluorometric analysis of extracellular crosslinking to Caco‐2 cell surface mediated by mTG and TG2 (*n* = 3). (D) Confocal microscopy analysis of extracellular crosslinking by mTG and TG2. Mean fluorescence intensity of transamidated 5BP (488 nm channel) was normalized to nuclei staining (405 channel) (*n* = 5). Scale = 20 µm. All data are shown as mean ± SD. Statistical significance was tested with the Student's *t* test. * *p* < 0.05, ** *p* < 0.01. 5BP, 5‐biotinamidopentylamine; mTG, microbial transglutaminase; SD, standard deviation; TG2, transglutaminase 2.

Next, we investigated whether mTG and TG2 are able to crosslink the transglutaminase substrate 5BP to the cell surface of Caco‐2 cells. First, we confirmed that the used concentrations of both transglutaminases did not affect cell viability (Figure S1). Crosslinking of 5BP by the cell surface TG2 of unpermeabilized cells was present at a low amount (Figure 3A, B). Addition of both transglutaminases resulted in a significant crosslinking of 5BP to the cell surface as staining of the substrate at the cell borders (Figure 3A–D). Again, mTG had a higher transamidation activity compared to TG2 (Figure 3A–D). Addition of mTG‐blocker and ERW1041 reduced crosslinking of 5BP at a dose‐dependent manner (Figure 3A–D).

### 3.4 mTG Increases Cellular Uptake of Fluorochrome‐Conjugated Gliadin Peptides

**FIGURE 4:**
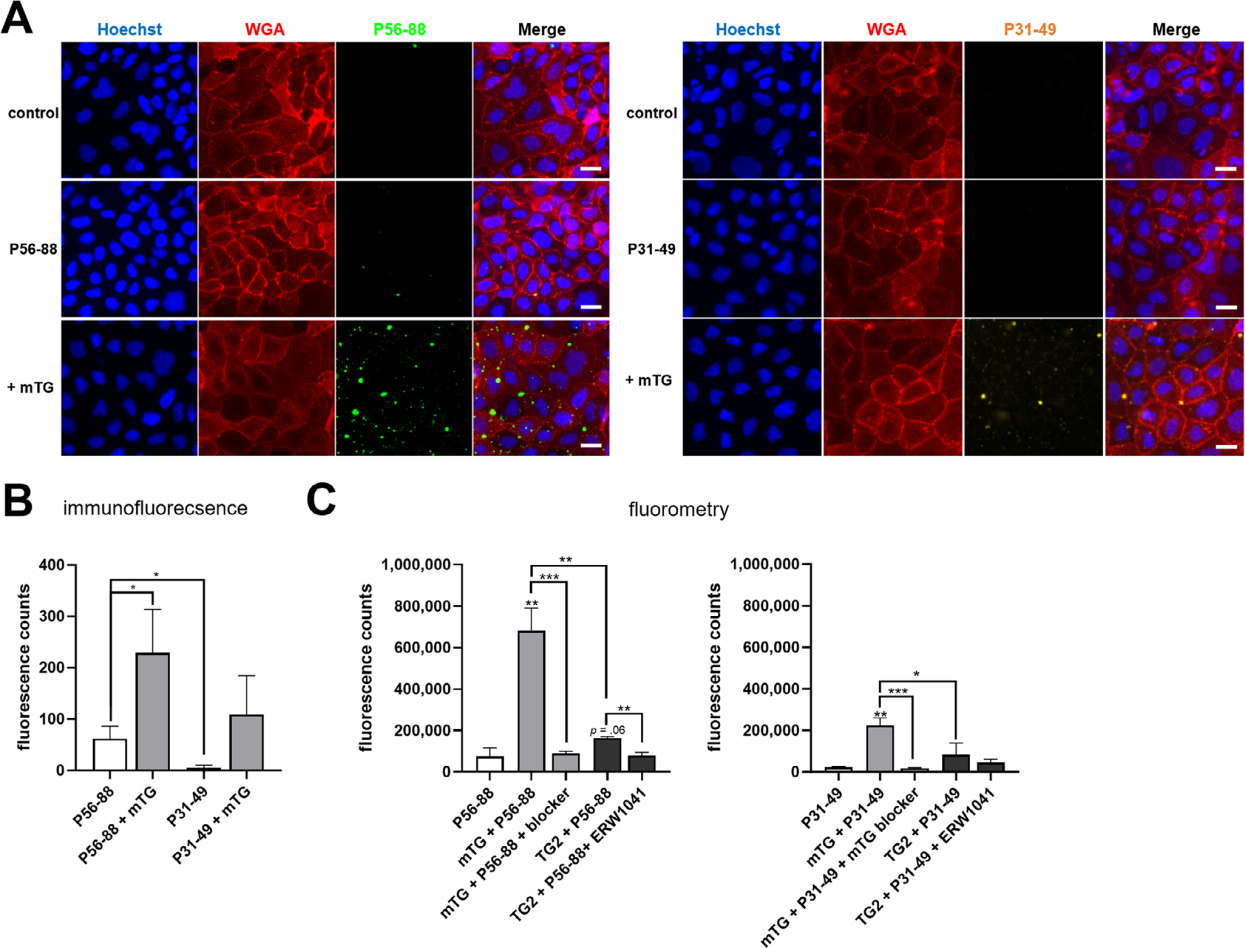
Epithelial uptake of 5‐FAM‐conjugated P56‐88 and P31‐49 is enhanced by mTG. (A) Representative immunofluorescence microscopy images showing the epithelial uptake of P56‐88 and P31‐49 in the presence of mTG. (B) Analysis of immunofluorescence microscopy images (*n* = 3, with five images per condition). (C) Fluorometric quantitation of the uptake of P56‐88 and P31‐49 in the presence of mTG and TG2 and their corresponding inhibitors (*n* = 6). Scale = 20 µm. All data are shown as mean ± SD. Statistical significance was tested with the Student's *t* test (with Welch correction where appropriate). * *p* < 0.05, ** *p* < 0.01, *** *p* < 0.001. 5‐FAM, 5‐carboxyfluorescein; mTG, microbial transglutaminase; SD, standard deviation; TG2, transglutaminase 2; WGA, wheat germ agglutinin.

To further investigate the influence of transglutaminases on gliadin peptide transport, Caco‐2 cell monolayers were incubated for 1 h with 5‐FAM‐conjugated gliadin peptides P56‐88 and P31‐49 in the presence of mTG. Significantly higher amounts of the potentially immunogenic gliadin peptide P56‐88 were taken up compared to the “toxic” gliadin peptide P31‐49 (Figure 4A, B). Addition of mTG significantly increased the cellular uptake of both peptides (Figure 4A, B). This observation was confirmed by fluorometry, where mTG enhanced the cellular uptake of both gliadin peptides by nearly 10 times (Figure 4C). In contrast, incubation with TG2 only resulted in a trend for an increased uptake of P56‐88 and P31‐49 (Figure 4C).

### 3.5 mTG Increases the Intracellular Uptake of Biotinylated Gliadin Peptides

**FIGURE 5:**
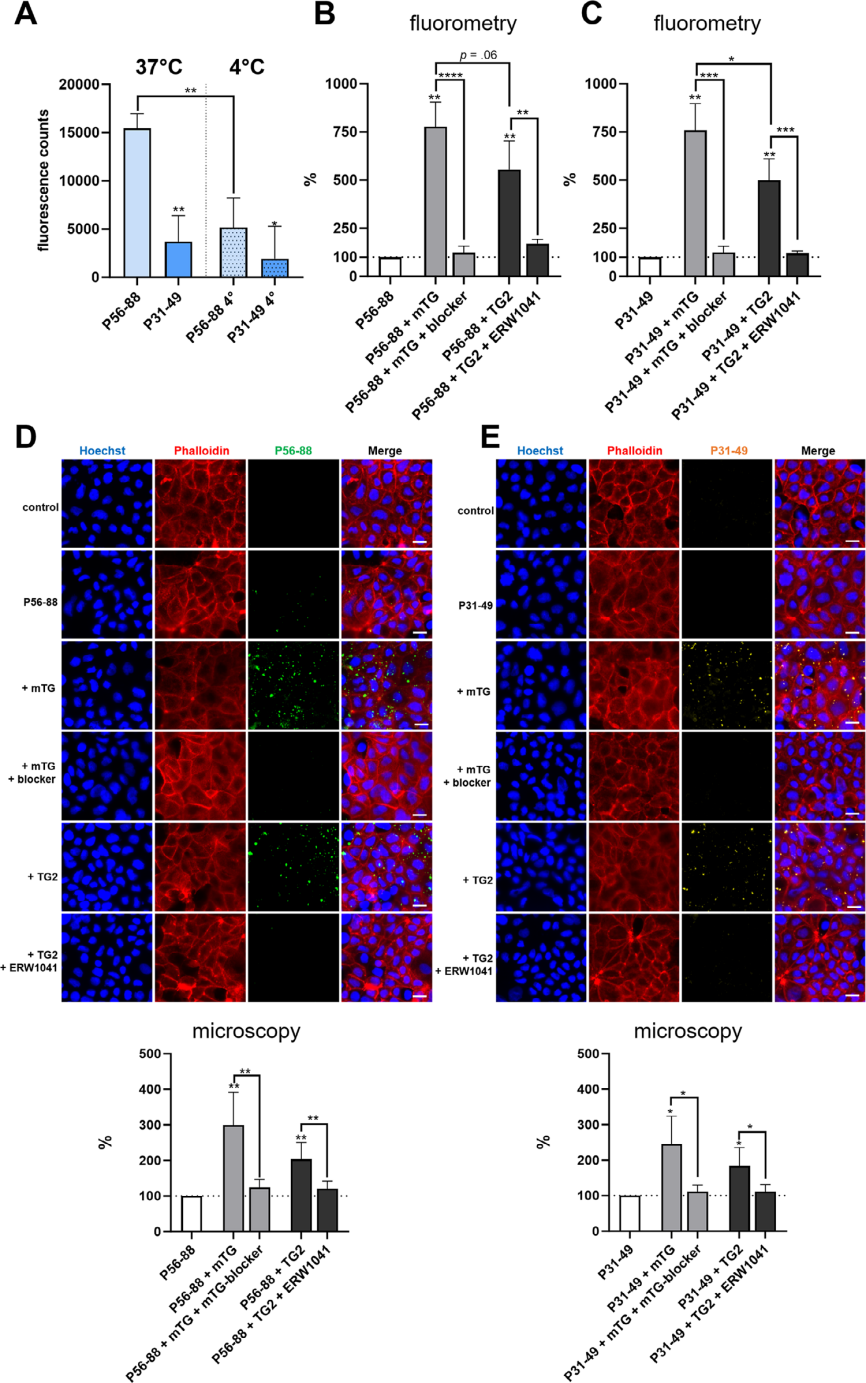
Cellular uptake of biotin‐conjugated P56‐88 and P31‐49 is enhanced by mTG. (A) Fluorometric analysis of gliadin peptide uptake by Caco‐2 cell monolayers at 37 and 4°C (*n* = 3). (B, C) Fluorometric analysis of P56‐88 (B) and P31‐49 (C) uptake in the presence of mTG and TG2 (*n* = 4). (D, E) Immunofluorescence microscopy analysis of P56‐88 (D) and P31‐49 (E) uptake in the presence of mTG and TG2 (*n* = 5, with five images per condition). Scale = 20 µm. All data are shown as mean ± SD. Statistical significance was tested with the Student's *t* test (with Welch correction where appropriate). * *p* < 0.05, ** *p* < 0.01, *** *p* < 0.001, **** *p* < 0.0001. mTG, microbial transglutaminase; SD, standard deviation; TG2, transglutaminase 2.

We used biotin‐conjugated P56‐88 and P31‐49 to further characterize the influence of mTG and TG2 on the cellular uptake and localization of both gliadin peptides. Fluorometric analysis on confluent Caco‐2 cell monolayers revealed a significant epithelial uptake of P56‐88. In contrast, P31‐49 was only taken up in a small amount, confirming our previous observations with 5‐FAM‐conjugated gliadin peptides (Figure 5A). We then performed the incubation at 4°C, which significantly reduced peptide uptake of P56‐88 and P31‐49 by about 75% and 50%, indicating a substantial role of endocytosis (Figure 5A). mTG significantly increased the uptake of P56‐88 and P31‐49 by about 7.5 times (Figure 5B). Incubation with TG2 also increased the uptake of both gliadin peptides but to a lower extent (by about five times) compared to mTG (Figure 5C). The addition of both inhibitors, mTG blocker and ERW1041, reduced transglutaminase‐mediated gliadin uptake to the baseline level (Figure 5B, C).

Using immunofluorescence microscopy, we confirmed our data from the fluorometry experiments. Baseline epithelial uptake of P31‐49 was lower compared to P56‐88 (131% ± 13%, *p* = 0.05, Figure 5D, E). mTG and TG2 enhanced the cellular uptake of P56‐88 by 299% ± 92% and 204% ± 47%, respectively. Addition of the specific inhibitors mTG blocker and ERW1041 reduced cellular peptide uptake to the baseline level (Figure 5D). The uptake of P31‐49 was also significantly increased by both transglutaminases (246% ± 79% and 184% ± 84%), and this effect was abolished by using the specific inhibitors (Figure 5E).

### 3.6 mTG Increases Cell Surface Crosslinking of Biotinylated Gliadin Peptides P56‐88 and P31‐49

**FIGURE 6:**
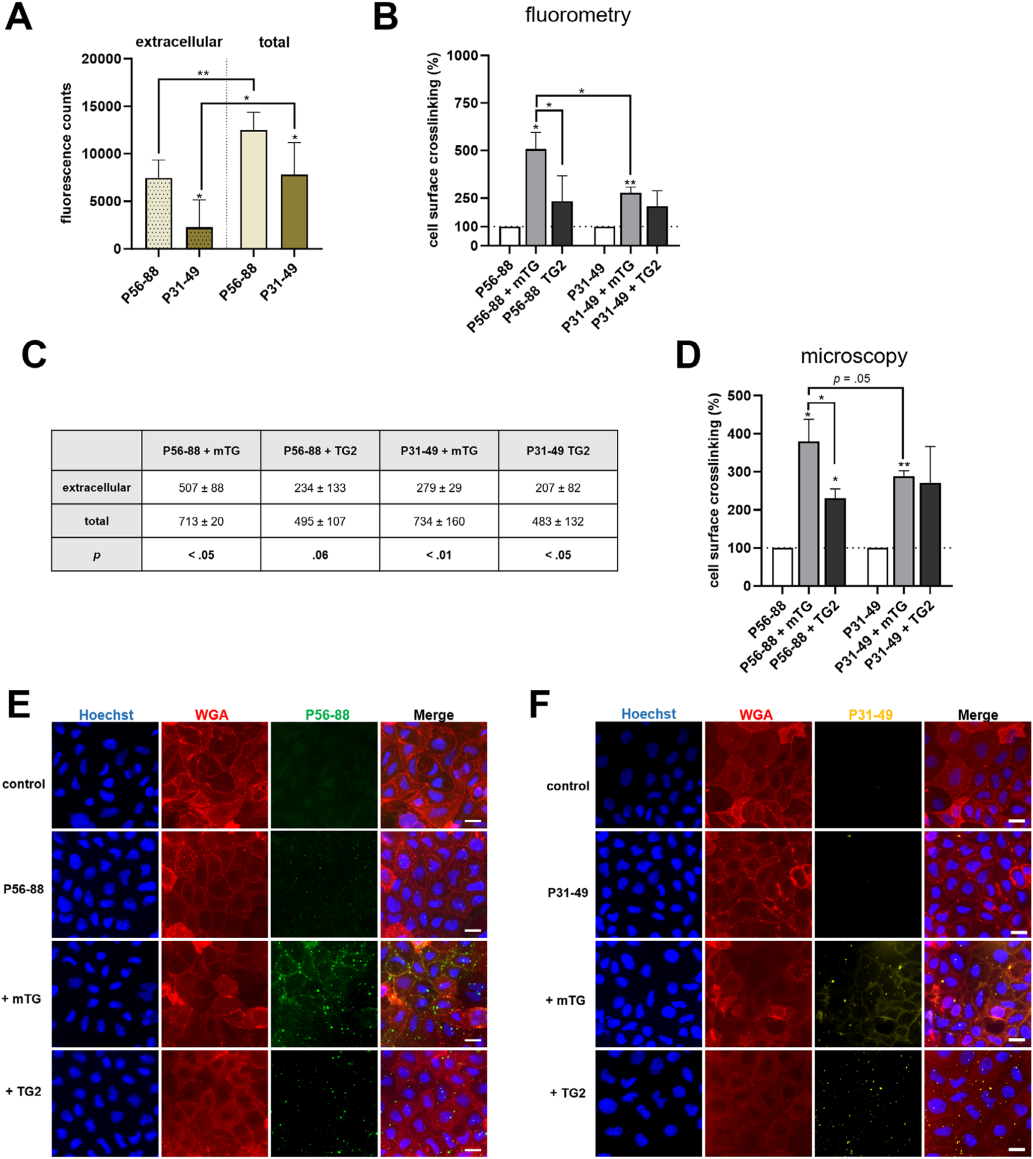
mTG crosslinks gliadin peptides P56‐88 and P31‐49 to the cell surface of Caco‐2 cells. (A) Fluorometric analysis of total (after permeabilization) and extracellular (without permeabilization) gliadin peptide uptake (*n* = 3). (B) Fluorometric analysis of extracellular crosslinking of P56‐88 and P31‐49 by transglutaminases (*n* = 3). (C) Comparison of fluorometric detection of P56‐88 and P31‐49 in the presence of transglutaminases without (extracellular) and with (total) permeabilization (*n* = 3). (D–F) Immunofluorescence microscopical investigation of extracellular crosslinking of P56‐88 and P31‐49 by mTG and TG2 (*n* = 5 with five images per condition). Scale = 20 µm. All data are shown as mean ± SD. Statistical significance was tested with the Student's *t* test (with Welch correction where appropriate). * *p* < 0.05, ** *p* < 0.01. mTG, microbial transglutaminase; SD, standard deviation; TG2, transglutaminase 2; WGA, wheat germ agglutinin.

To analyze whether the biotinylated peptides were localized in the intra‐ or extracellular compartment, we stained P56‐88 and P31‐49 using streptavidin‐Alexa Fluor488 without permeabilization. In unpermeabilized Caco‐2 cells, the peptide‐derived fluorescent signal was significantly lower compared to the signal after permeabilization (Figure 6A). This indicated that the majority of both peptides was transported to the intracellular compartment after 1 h of incubation. Addition of mTG markedly enhanced extracellular labeling with both gliadin peptides, but the effect was significantly higher for P56‐88 (Figure 6B, C). TG2 also increased cell surface crosslinking of both gliadin peptides, but to a lesser extent compared to mTG (Figure 6B, E, F).

To further visualize the crosslinking of P56‐88 and P31‐49 to the cell surface, we performed immunofluorescence microscopy, confirming the results of the fluorometric assays (Figure 6D). In detail, we only observed a very low amount of extracellular gliadin peptides, especially when compared to the data we obtained for the intracellular uptake of both peptides (Figures 5D, E and 6E, F). Addition of mTG caused a specific fluorescence pattern at the cell boundaries with a similar configuration as observed for the extracellular crosslinking of 5BP (Figures 3C, D and 6E, F). Treatment with TG2, however, revealed a lower grade of crosslinking, especially for P56‐88 (Figure 6D–F).

### 3.7 mTG Increases the Epithelial Passage of P56‐88 in the Duodenal Mucosa

**FIGURE 7:**
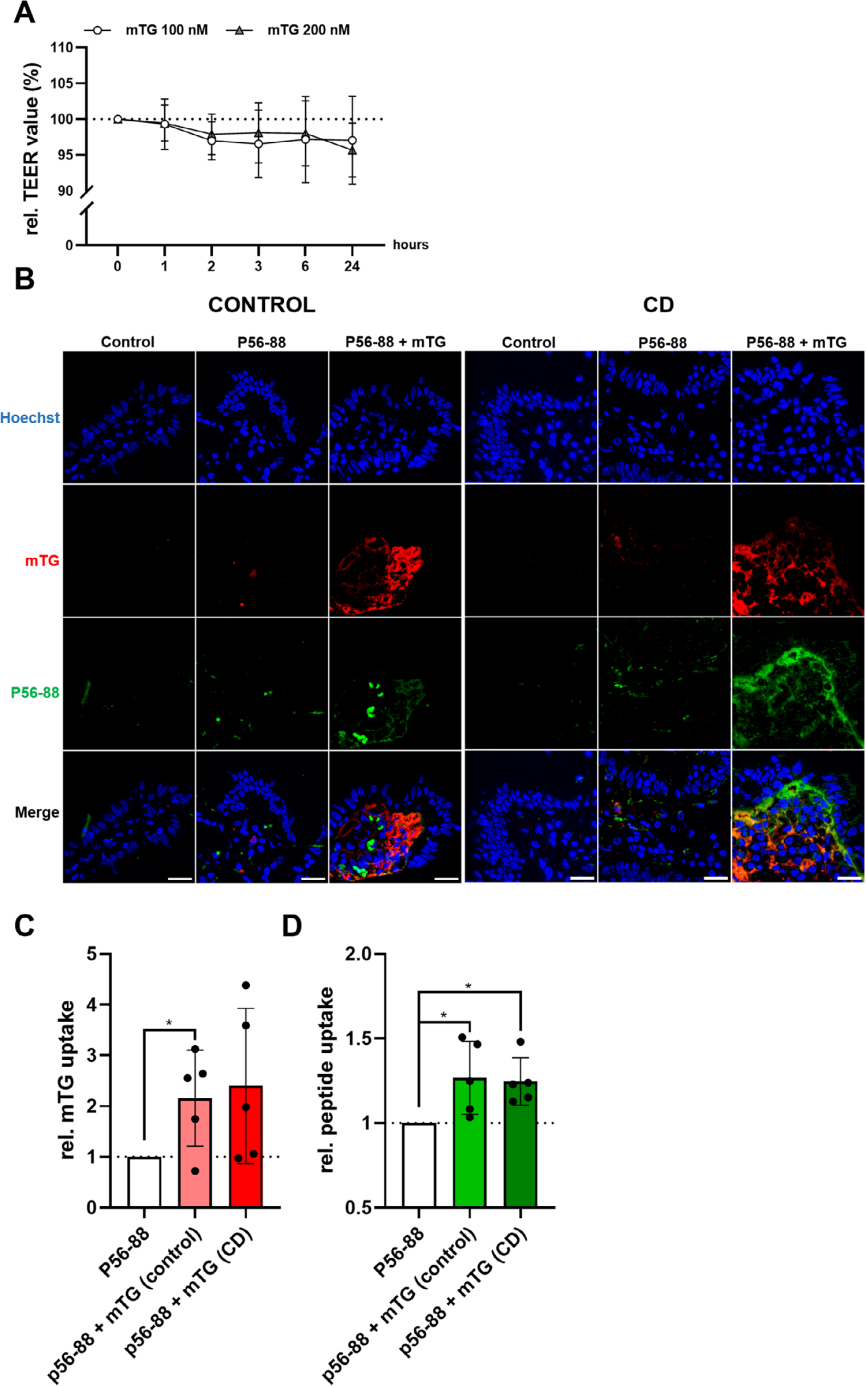
mTG increases epithelial transport of P56‐88 in human duodenal biopsies. (A) No effect of mTG on the transepithelial electrical resistance (TEER) in Caco‐2 cell monolayers (*n* = 3). (B) Representative immunofluorescence microscopy images of duodenal biopsies incubated with biotinylated P56‐88 ± mTG. (C) Immunofluorescence microscopical quantitation of the staining of mTG in the duodenal *lamina propria* (three images per condition). (D) Immunofluorescence microscopical quantitation of the staining of P56‐88 in the duodenal *lamina propria* (three images per condition). Scale = 20 µm. All data are shown as mean ± SD. Statistical significance was tested with Student's *t* test (with Welch correction where appropriate). * *p* < 0.05. mTG, microbial transglutaminase; SD, standard deviation.

Finally, we investigated the effect of mTG on the mucosal uptake of P56‐88 in the human duodenum. First, we addressed the question whether mTG affects intestinal epithelial barrier integrity in confluent Caco‐2 cell monolayers. At concentrations of 100 and 200 nM, mTG did not alter the TEER in this model over a period of 24 h (Figure 7A). For an ex vivo‐incubation using P56‐88 with and without mTG, human duodenal biopsies were placed in one layer between circular aperture sliders to ensure apical uptake. After 30 min, mTG translocated to the intestinal *lamina propria* of control and CD biopsies (Figure 7B, C), however, with a high interindividual variability in both groups. We did not observe any staining of mTG in the duodenal epithelium (Figure 7B). P56‐88 was transported to the *lamina propria* of both control and CD biopsies in the absence of mTG, but only to a very small extent (Figure 7B). The simultaneous incubation of P56‐88 and mTG resulted in a significantly increased uptake of P56‐88 that reached the duodenal *lamina propria* in both groups (controls 1.27 ± 0.22; CD 1.25 ± 0.14, Figure 7C). Neither the uptake of mTG nor the uptake of P56‐88 differed between control and CD patients (Figure 7C, D).

## 4 DISCUSSION

An exposure to gliadin is necessary, but not sufficient in the majority of genetically predisposed individuals to trigger the development of CD [1]. Currently, there is still a debate regarding dietary and microbial factors that might contribute to CD pathogenesis. One of the environmental factors that was suggested is mTG. In the past, studies mostly focused on the ability of mTG to deamidate specific glutamine residues of the 33‐mer gliadin peptide. Dekking et al. have shown that mTG mimics the deamidation pattern of TG2 in gliadin peptides [17]. Skovbjerg et al. confirmed this observation and further reported that other transglutaminases (e.g., Factor XIIIa) did not deamidate the corresponding gliadin peptide [25]. Even though the deamidation rate was lower compared to TG2, significant amounts of the immunogenic epitope PELP were produced after treatment with mTG [25, 34]. In our study, we aimed to compare the interaction of mTG and TG2 with gliadin peptides apart from deamidation. We demonstrated that mTG has a much higher crosslinking affinity not only for the potentially immunogenic gliadin peptides P56‐68 and P56‐88 but also for other peptides, including the deamidated immunogenic peptide (P57‐73E), as well as for P31‐49 and P229‐246. In contrast to TG2, mTG did not crosslink gliadin peptides among each other, but transamidated the peptides to other structures, including the cell surface of Caco‐2 cells. The crosslinking of different gliadin peptides by mTG to other food components might thus induce so‐called neo‐epitopes that may have a high immunogenic potential for instance for people carrying the genetic predisposition for CD.

mTG also increased the epithelial uptake of fluorochrome‐ and biotin‐conjugated gliadin peptides P56‐88 and P31‐49 by nearly 10 times and was much more potent than TG2. The baseline epithelial uptake of P31‐49 was low, whereas higher amounts of P56‐88 were found in Caco‐2 cells after treatment with equimolar concentrations. Incubation at 4°C reduced the epithelial uptake of both gliadin peptides indicating an endocytotic process, which has already been described before [35]. Simultaneous incubation of P56‐88 and P31‐49 with mTG only resulted in comparatively low amounts of cell surface‐bound peptides, suggesting that the addition of transglutaminases promoted the intracellular uptake.

Finally, we addressed the question, whether mTG increases the epithelial passage of P56‐88 in human duodenal tissue. Using a directed ex vivo incubation, we found significant uptake of mTG in control and CD biopsies after 30 min. At this time point, mTG had already reached the intestinal *lamina propria*. This observation is in line with our previous findings, where we demonstrated the localization of mTG in the duodenal *lamina propria* after 60 min of incubation by immunofluorescence and electron microscopy [36]. As described before, we did not observe mTG within the epithelium. However, this might be explained by the lower sensitivity of immunofluorescence microscopy compared to electron microscopy [36].

In addition, we demonstrated a significantly increased uptake of P56‐88 to the *lamina propria* in the presence of mTG. This observation might be of high importance since TG2‐mediated enzymatic modification takes place in this compartment [37]. Thus, mTG may promote the production of immunogenic gliadin peptides by increasing the mucosal passage of P56‐88 and its subsequent modification by TG2 in this compartment. Since mTG did not alter the TEER of Caco‐2 cells, it seems likely that mTG increases the transcellular and not the paracellular passage of gliadin peptides. Altogether, our data indicate that mTG either derived from processed food products or the intestinal microbiota might play a detrimental role in CD pathogenesis. Regarding the food industry, mTG is used to improve textural properties especially of restructured meat products, but its application has also been described for a multitude of other foods, including gluten‐free bread as well [26, 28]. mTG‐treated products are typically subjected to heat treatment, effectively deactivating the enzyme before the food reaches consumers. However, for some mTG‐treated food items, heat processing is not applied (restructured meat/fish products, surimi, vegetarian/vegan meat analogues), raising the possibility that active mTG may be ingested. Furthermore, nonenzymatic and enzymatic digestion in the upper gastrointestinal tract might affect the interaction between mTG and gliadin peptides. The concentrations of mTG used in our study ranged from 10 nM to 1 µM (0.4–40 µg/mL), which is considerably below the concentrations that are used in the food industry (up to 1% or 100–10 mg/mL) [26, 27]. Our findings demonstrate that mTG has a very high reactivity and crosslinks all investigated gliadin peptides. The presence of active mTG, gliadin, and other proteins in a food mixture might favor the creation of immunogenic neo‐epitopes, which might induce the development of CD in genetically predisposed individuals. On the contrary, there is some evidence from in vitro data that mTG‐treatment theoretically can mask potentially immunogenic epitopes from gliadin peptides by crosslinking. Even though some studies have demonstrated a reduced affinity of antigliadin‐ and antideamidated‐gliadin‐antibodies after mTG‐treatment, potentially immunogenic epitopes were not completely diminished [38, 39, 40, 41]. In addition, clinical trials using mTG‐treated rusks or bread resulted in high dropout rates (30%–50%) due to CD‐related symptoms [38, 42]. Whether mTG is released into the gut lumen by the intestinal microbiota and might thus encounter food‐derived gliadin peptides in an active state will be addressed in future studies.

In summary, our data highlight the strong interaction of mTG and gliadin, which increased the transcellular uptake of potentially detrimental gliadin peptides. The uncritical usage of mTG in food products might thus pose a potential threat for consumers, especially those who are at risk to develop CD.

## Conflicts of Interest

The authors declare no conflicts of interest.

## Supporting information




**Supporting File 1**: mnfr70197‐supp‐0001‐SuppMat.docx

**Supporting File 2**: mnfr70197‐supp‐0002‐FigureS1.pdf

## Data Availability

The data that support the findings of this study are available from the corresponding author upon reasonable request.
