# Supplementary material for: Microbial Transglutaminase Increases Uptake and Translocation of Gliadin Peptides in the Human Intestinal Epithelium
Source: Mol Nutr Food Res. 2025 Aug 11;69(21):e70197. doi: 10.1002/mnfr.70197 (PMC12581733; doi:10.1002/mnfr.70197)
Supplement: Supplementary file 1 — Supporting File 1: mnfr70197‐supp‐0001‐SuppMat.docx [file MNFR-69-e70197-s001.docx]

**SUPPLEMENTARY MATERIAL - METHODS**

**Cell viability of Caco-2 cells**

Cells were incubated with mTG or TG2 at the indicated concentrations for 24 hours in culture medium. Incubation with PrestoBlue HS, diluted 1:10 in medium was performed for 2 hours at 37°C. Fluorometric examination was conducted using a Clariostar Plus microplate reader.

**Epithelial transport of gliadin peptides in the presence of mTG**

After incubation with 5-FAM-conjugated peptides, cells were washed with PBS and fluorescence was measured at 488 nm using a Clariostar Plus (BMG Labtech) microplate reader. After incubation with biotinylated gliadin peptides, cells were fixed with 4 % paraformaldehyde and permeabilized where indicated with 0.5 % triton. After blocking with 5 % BSA, biotinylated P56-88 and P31-49 were detected by streptavidin-Alexa Fluor 488 (2 µg/ml, Thermo Fisher Scientific) and fluorometric quantitation was performed as described before.

For immunofluorescence microscopy, cells were seeded on polymer-covered µ-slides (80826, Ibidi, Gräfelfing, Germany). After incubation with 5-FAM- or biotin-conjugated peptides, cells were fixed and permeabilized where indicated as described before. Cell membranes were stained with CF555 wheat germ agglutinin (unpermeabilized cells, WGA, 1:250, 29076, Biotium Inc, Fremont, CA, USA) or phalloidin-rhodamine (permeabilized cells, 1:100, R415, Thermo Fisher Scientific) and nuclei were stained with Hoechst dye.

After incubation with 5-FAM-conjugated peptides, cells were washed two times with cold PBS and fluorescence was measured at 488 nm using a Clariostar Plus (BMG Labtech) microplate reader. For microscopic quantitation, cells were seeded on polymer-covered µ-slides (80826, Ibidi, Gräfelfing, Germany). After incubation, cells were washed with cold PBS and fixed with 4 % paraformaldehyde. The cell membrane was stained with CF555 wheat germ agglutinin (WGA, 1:250, 29076, Biotium Inc, Fremont, CA, USA) and nuclei were stained with Hoechst dye. Microscopy was performed using a Leica DMi8 fluorescence microscope (Leica, Wetzlar, Germany) and a 20x (NA 0.8) objective.
