# Supplementary figures and images for: Microbial Transglutaminase Increases Uptake and Translocation of Gliadin Peptides in the Human Intestinal Epithelium
Source: Mol Nutr Food Res. 2025 Aug 11;69(21):e70197. doi: 10.1002/mnfr.70197 (PMC12581733; doi:10.1002/mnfr.70197)

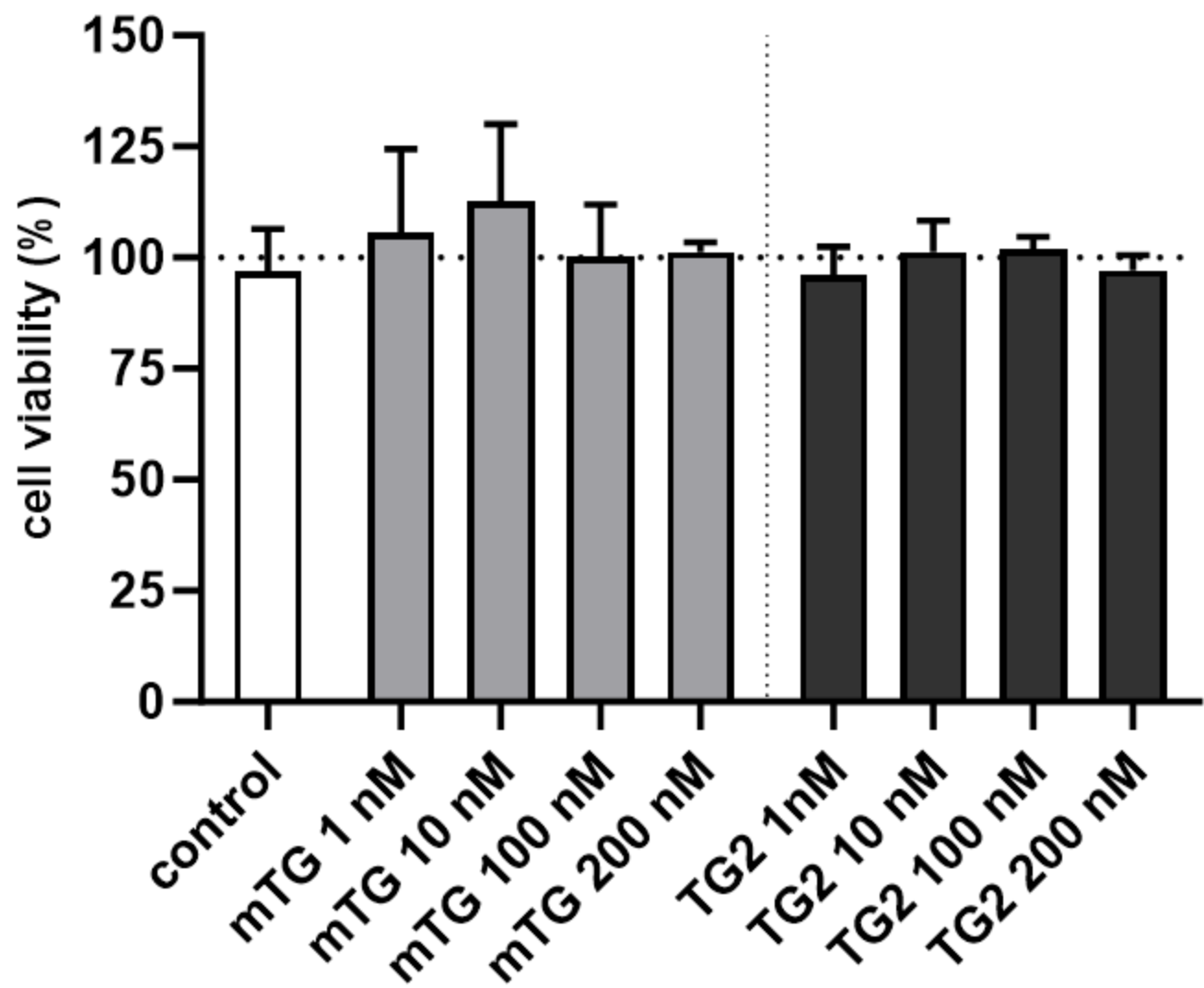

Supplement: Supplementary file 2 — Supporting File 2: mnfr70197‐supp‐0002‐FigureS1.pdf [file MNFR-69-e70197-s002.pdf]
